# Recent advances in the understanding and management of IgA nephropathy

**DOI:** 10.12688/f1000research.7352.1

**Published:** 2016-02-11

**Authors:** Kar Neng Lai, Joseph C.K. Leung, Sydney C.W. Tang

**Affiliations:** 1 Nephrology Department, Hong Kong Sanatorium and Hospital, Happy Valley, Hong Kong; 2 Nephrology Division Department of Medicine, University of Hong Kong, Queen Mary Hospital, Hong Kong

**Keywords:** IgA nephropathy, glomerulonephritis, end-stage renal disease, genetics of IgAN, Familial IgAN, treatment IgAn

## Abstract

Since its first description in 1968, IgA nephropathy has remained the most common form of primary glomerulonephritis leading to chronic kidney disease in developed countries. The clinical progression varies, and consequent end-stage renal disease occurs in 30% to 40% of patients 20 to 30 years after the first clinical presentation. Current data implicate overproduction of aberrantly glycosylated IgA1 as being pivotal in the induction of renal injury. Effective and specific treatment is still lacking, and new therapeutic approaches will be developed after better understanding the disease pathogenesis.

## Introduction

IgA nephropathy (IgAN) remains the most common primary glomerulonephritis worldwide. Other than diabetic nephropathy, IgAN remains the next important health-care issue in nephrology as it often affects young adults and the nephropathy pursues a slow but relentless clinical course. Consequent end-stage renal disease (ESRD) occurs in 30% to 40% of patients within 20 to 30 years after clinical presentation. The kidney is a target of injury in IgAN, yet the primary defect originates from a systemic aberrant glycosylation of *O*-linked glycans in the hinge region of IgA1, resulting in increased serum levels of galactose-deficient IgA1 (Gd-IgA1). As the immunochemical abnormality of IgA is not corrected by renal transplantation, not surprisingly IgAN can frequently recur in allograft. Effective and specific treatment for IgAN is still lacking.

Serum Gd-IgA1 levels are heritable in a dominant pattern with reduced penetrance, although most patients’ relatives who have high serum levels of Gd-IgA1 do not exhibit clinical manifestations of renal injury. IgAN may occur in either sporadic or familial pattern. Familial IgAN may have a poorer prognosis with an increased risk of progression to renal failure, but this is controversial. Patients with familial IgAN have increased serum levels of galactose-deficient macromolecular IgA1 as compared with patients with sporadic IgAN. Macromolecular IgA1 isolated from patients with familial IgAN has enhanced binding to mesangial cells *in vitro*. These observations support the notion that genetic factors are involved in the pathogenesis of familial, as well as sporadic, IgAN, and risk factors of multiple candidate genes have been identified in different ethnic groups. The goal of this review is to present the genetic data discovered in the last decade, and discuss the treatment options in IgAN.

## Genetic data

Epidemiological data support a strong genetic contribution to IgAN ^1^. First, there are significant geographic and ethnic differences in the prevalence of IgAN; the highest frequency is in East Asians, and it is relatively common in Mediterranean countries and very uncommon in individuals of African ancestry ^2, 3^. Second, IgA1 glycosylation defects exhibit high heritability in relatives of familial IgAN patients ^4– 6^. Familial IgAN presenting as autosomal dominant transmission with incomplete penetrance has been well recognized ^1^. Despite numerous efforts at gene mapping by using linkage approaches, Mendelian defects responsible for familial IgAN remain elusive unless large families are available for study. Earlier studies of IgAN families revealed four linked loci.

6q22-q23 ( *IGAN1*) and 3p24-p23 loci were the first loci identified in linkage analysis of IgAN with a logarithm of the ratio of odds (LOD) score of 5.6 ^7^. The study analyzed 24 Italian and six American families, suggesting another locus at 3p24-p23 with a maximum LOD score of 2.8. Next, 2q36 locus was revealed in a four-generation Canadian family of German-Austrian origin with 14 affected and 11 unaffected members ^8^. The pedigree is consistent with autosomal dominant inheritance. Parametric and non-parametric linkage analysis produced significant LOD scores according to standard criteria for Mendelian disease. Finally, a European IgAN consortium identified suggestive loci at 4q26-q31 (LOD score of 1.8) and 17q12-q22 (LOD score of 2.6) in 22 Italian families with 59 affected and 127 unaffected members. The loci were named *IGAN2* and *IGAN* ^9^. Intriguingly, these four loci have not been revealed in familial IgAN of other ethnicity ^10^.

The genome-wide association study (GWAS) approach has emerged as a powerful alternative to family-based studies for complex traits and has been successfully applied to IgAN. The first GWAS for IgAN based on European patients ^11^ was shortly followed by two larger studies performed in Chinese cohorts of Hans ethnicity ^12, 13^. Notably, all three studies consisted of a relatively small discovery sample of 3,000 IgAN cases. Seven independent risk loci with genome-wide significance ( *P* < 5 × 10 ^-8^) were identified and these loci cumulatively explained approximately 5% of the overall disease variance.

The IgAN risk allele frequencies correlate well with disease epidemiology ^3^. Notably, East Asians with the highest prevalence of IgAN carry the highest average number of risk alleles, but IgAN is less common in Africans with the lowest burden of risk alleles. IgAN as a leading cause of ESRD is nearly 10-fold higher among US kidney patients with East Asian ancestry when compared with African Americans ^3^. Since over 85% of the existing GWAS discovery cohorts are ethnic Chinese, studies in other ethnicities (especially from Mediterranean countries and Australasia with moderate prevalence) are needed to understand genetic risk profiles among other populations.

Contrary to the genetic approaches using linkage studies, and GWAS that requires a large patient cohort of sporadic IgAN, Liu *et al.* ^14^ studied 10 IgAN families of Han Chinese ethnicity by using exome sequencing techniques. IgAN families are enriched in genetic components predisposing individuals to the development of this disorder. The technique of exome sequencing allows the interrogation of the whole exome to identify genes and gene variants that underlie both monogenic and complex diseases. Six deleterious variants in four genes associated with familial IgAN were discovered. Of interest is the association of *DEFA* gene and the disease susceptibility in both sporadic and familial IgAN of Han Chinese ethnicity with different mutations ^14, 15^.

Through careful analysis and annotation of the detected loci, several causal candidate genes have been prioritized, linking pathways involved in the pathogenesis of IgAN. The implicated pathways include (i) the antigen-processing and presentation pathway (three loci on chromosome 6p21 in the major histocompatibility complex [MHC] region), (ii) the mucosal immunity pathway (chromosomes 22q12 *HORMAD2* locus, 8p23 α-defensin [ *DEFA*] locus, and 17p13 *TNFSF13* locus), and (iii) the alternative complement pathway (chromosome 1q32 complement factor H [ *CFH*] locus) ^11– 13^.

### (i) Antigen-processing and presentation pathway

All three GWASs of IgAN identified strong signals within the MHC region with three distinct susceptibility loci on chromosome 6p21: *HLA-DRB1/DQB1*, *HLA-DPB1/DPB2*, and *TAP1/PSMB9*. *HLA-DRB1-DQA1* and *-DQB1* genes carry the strongest association, and the DQB1*0602-DRB1*1501 haplotype confers a highly protective effect ^12^.

The second distinct MHC locus was centered over the region of the *HLA-DPA1*, *-DPB1*, and *-DPB2* genes (also encoding MHC-II molecules), but the causal variant at this locus and its involvement in IgAN are still not known. The third MHC locus contained the *TAP1, TAP2, PSMB8*, and *PSMB9* genes. These genes play an important role in modulation of cytokine production and cytotoxic T-cell response through antigen processing for presentation by MHC-I molecules.

### (ii) Mucosal immunity and regulation of IgA production

The clinical characteristic of synpharyngitic macroscopic hematuria led to the hypothesis that defects in the regulation of local IgA response or abnormal mucosal antigen handling (or both) may trigger IgAN ^16^.

APRIL, a proliferation-inducing ligand, is the molecule involved in T cell-independent generation of IgA-secreting plasma cells as well as in the IgA1 to IgA2 class switching. A GWAS locus on chromosome 17p13 contains *TNFSF13* that encodes APRIL. Serum levels of APRIL are elevated in some patients with IgAN ^17^, and raised total serum IgA occurs with the 17p23 risk variant ^12^. Overexpression of B-cell activation factor (BAFF), a related molecule with overlapping functions and receptors with APRIL, results in mesangial IgA deposits in mice ^17^. A recent study from Japan showed that treatment of the newly developed grouped ddY (gddY) mice with anti-APRIL antibody reduced serum IgA levels, glomerular IgA deposition, albuminuria, and renal damage ^18^. These data suggest that APRIL and BAFF signaling may be involved in the pathogenesis of IgAN and that both may be potential therapeutic targets.

A locus on chromosome 22q12 also influences serum IgA levels and encompasses several genes, including the IL-6 family-encoding genes *LIF* and *OSM* ^11^.

The *DEFA* gene cluster on chromosome 8p23 is the third IgAN GWAS locus implicated in mucosal immunity. The α-defensin gene family encodes small, structurally related peptides that are secreted at mucosal surfaces with microbiocidal and chemoattractant properties ^19^. α-defensin 1, 3, and 4 (encoded by *DEFA1*, *DEFA3*, and *DEFA4*) are synthesized in neutrophils, whereas α-defensin 5 and 6 ( *DEFA5* and *DEFA6*) are constitutively released by the intestinal Paneth cells into the gut lumen. It remains unclear whether the IgAN risk allele in this region confers a risk haplotype due to excessive copies of *DEFA1/3* genes or variants of *DEFA5/6* genes.

Lately, six new genome-wide significant associations—four in *ITGAM*, *ITGAX*, *VAV3*, and *CARD9* and two new independent signals at *HLA-DQB1* and *DEFA*—were identified in a GWAS examining 20,612 IgAN individuals of European and East Asian ancestry ^20^. Most loci are directly associated with either risk of inflammatory bowel disease or maintenance of the intestinal epithelial barrier and response to mucosal pathogens. A possible role for host-intestinal pathogen interactions in shaping the genetic landscape of IgAN has been proposed.

### (iii) Alternative complement pathway

A common deletion (deleting *CFHR3* and *CFHR1* genes), within the *CFH* locus on chromosome 1q32, was found to be protective against IgAN in a GWAS studying both European and Asian populations ^12^. The *CFH* gene encodes Factor H (FH) that regulates the alternative complement pathway. FH-related proteins (FHR1–5) are structurally similar to FH and are encoded by five genes ( *CFHR1-5*) residing within the same genomic region. Given the high level of sequence similarity between *CFH* and *CFHR*s, these genes are believed to have originated through segmental duplications and are prone to recurrent structural rearrangements. *CFHR3,1*Δ is the most common variant; allelic frequency ranges from 0% to 5% in East Asians to 20% in Europeans and up to 50% in some African populations ^21^. Each additional copy of *CFHR3,1*Δ reduces the risk of IgAN by approximately 40% ^12^.

## Treatment

### General

Patients with minor urine abnormalities and normal blood pressure and glomerular filtration rate (GFR) usually do well and require only periodic monitoring, such as biennial clinic visits. For other patients, the therapeutic options are limited and include non-specific treatment to reduce proteinuria by renin-angiotensin system (RAS) blockade and non-specific control of inflammation using fish oil and agents such as corticosteroids, cytotoxic agents, anti-metabolite, and immunomodulatory drugs.

### Conventional therapy

***Renin-angiotensin-aldosterone axis blockade.*** Evidence accumulated from 56 studies and 2,838 participants showed that only anti-hypertensive drugs—mostly angiotensin-converting enzyme inhibitor (ACEI) or angiotensin receptor blocker (ARB)—provided useful intervention mainly by reducing proteinuria ^22^. RAS blockers are often prescribed for patients with IgAN and proteinuria. In a meta-analysis of 585 patients from 11 randomized clinical trials (RCTs) ^23^, significant renoprotection and reduction of proteinuria were achieved with an ACEI or ARB versus control. The beneficial effects are promoted by concomitant dietary sodium and phosphate restriction. In addition, the efficacy of RAS blockade could be modified by ACE (I/D) gene polymorphisms such that, in the future, personalized medicine could be developed using pharmacogenomics data ^24^.

Aliskiren is an oral direct renin inhibitor that has a theoretical basis for fully suppressing the RAS as ACEI or ARB treatment leads to a reactive increase in plasma renin activity. So far, the only two trials from Hong Kong showed an anti-proteinuric effect on top of ACEI or ARB therapy ^25, 26^. Patients with more advanced chronic kidney disease are prone to developing hyperkalemia. Long-term outcomes have not been reported.

***Fish oil.*** The possible benefit of fish oil containing omega-3 polyunsaturated fatty acid in the treatment of IgAN rests on reducing intra-renal inflammation by mitigating inflammatory cytokines and eicosanoids. However, the published reports failed to show convincing benefits.

In the original Mayo Clinic multicenter study with 106 subjects ^27^, fewer patients randomly assigned for fish oil treatment reached the end-point of at least a 50% rise in serum creatinine. Notably, neither this original study nor a subsequent trial showed a reduction of proteinuria. Proteinuria is a key therapeutic target because it may itself cause renal injury, and its reduction correlates with preservation of renal function. A recent trial of 30 patients suggested that a RAS blocker combined with polyunsaturated fatty acids reduced proteinuria more than RAS blocker alone ^28^. The KDIGO (Kidney Disease: Improving Global Outcomes) 2012 Clinical Practice Guidelines ^29^ suggest optional use of fish oil in the treatment of patients with persistent proteinuria of more than 1 g/day, despite 3 to 6 months of optimized supportive care including ACEI or ARBs and blood pressure control. Yet, the long-term benefits on preventing ESRD are uncertain.

### Immunosuppressive therapy

As stated earlier, IgAN is an autoimmune kidney disease and hence immune modulation targeting the putative pathogenic pathways may alter the disease progression. To date, no medications have been approved by the US Food and Drug Administration specifically for IgAN. The availability of new agents with novel mechanisms and activities against the humoral immune response may allow targeted treatment. Herein, we examine the existing evidence for immunosuppressive therapy in IgAN.

***Corticosteroids.*** Since early 1980, corticosteroids were often prescribed to IgAN patients with moderate to severe persisting proteinuria (variably defined as more than 0.5 to 1.0 g/day lasting for at least 3 months). A meta-analysis of nine randomized controlled trials (including 536 patients with urinary protein excretion of more than 1 g/day and normal renal function) suggested that high-dose and short-term corticosteroid therapy produced significant renal protection but that low-dose, long-term corticosteroid use did not ^30^. The 2012 KDIGO Guidelines ^29^ recommend that patients with persistent proteinuria of more than 1 g/day despite adequate ACEI or ARB and blood pressure control and a GFR of more than 50 ml/min per 1.73 m ^2^ receive a 6-month course of steroid therapy. A significant knowledge gap thus existed because patients with an estimated GFR (eGFR) of 30 to 50 ml/min per 1.73 m ^2^ have been excluded from virtually all major clinical trials. A recent retrospective analysis of the European Validation Study of the Oxford Classification of IgAN (VALIGA) cohort of 1,147 patients (mostly white) may help to address this gap ^31^. In the propensity score analysis, adding corticosteroid to RAS blocker resulted in a better reduction of proteinuria, a slower rate of renal function decline, and increased renal survival in comparison with administering RAS blocker alone in two groups of patients with a similar risk profile of progression. These benefits extended to 115 patients with an eGFR of less than 50 ml/min per 1.73 m ^2^, and the benefits increased proportionally with the level of baseline proteinuria. However, the study is limited by its retrospective nature, unknown corticosteroid dosing regimens, frequent combination of corticosteroids with other immunosuppressive therapies, the potential for unmeasured and selection bias, and the potential for selection of patients by the participating center ^32^.

Two new trials were conducted to further address the therapeutic value of conventional corticosteroid. STOP-IgAN is a German trial that randomly assigned adults with an eGFR of at least 30 ml/min per 1.73 m ^2^ and persistent proteinuria of more than 0.75 g per day despite 6 months of supportive care (in particular, blockade of the RAS to a target blood pressure of less than 125/75 mm Hg) to receive supportive care alone or supportive care plus immunosuppression (corticosteroids alone if eGFR was 60 to 89 ml/min per 1.73 m ^2^ or in combination with cyclophosphamide for the initial 3 months, followed by azathioprine if eGFR was 30 to 59 ml/min per 1.73 m ^2^) ^33^. During the run-in phase completed by 309 of 337 patients, proteinuria decreased to less than 0.75 g per day in 30% of the subjects, who then became ineligible for random assignment. Of 154 patients who underwent random assignment and completed 3 years of treatment, more patients in the immunosuppression group achieved full clinical remission (urine protein/creatinine of less than 0.2 g/g and reduction in eGFR of less than 5 ml/min per 1.73 m ^2^), but there was no significant difference in the annual decline in eGFR between the two groups. Patients in the immunosuppression group had a significantly lower mean proteinuria level than those in the supportive-care group at 12 months after random assignment, but this difference disappeared at 36 months. The major conclusion was that the addition of immunosuppressive therapy to intensive supportive care did not significantly improve the outcome and may increase adverse effects. The study is limited by its open-label nature, relatively short duration of follow-up for the end-point of renal deterioration, the lack of histologic stratification for inclusion, and the questionable design of assigning steroid monotherapy to patients with an eGFR of more than 60 ml/min per 1.73 m ^2^ and the addition of cyclophosphamide followed by azathioprine to patients with even lower eGFR. Finally, the findings could be conflicted by a relatively high proportion of subjects who were given combined ACEi and ARB treatment.

TESTING is another large multicenter, double-blinded, randomized, placebo-controlled trial in progress. TESTING started recruitment in 2012 internationally to investigate the efficacy of oral methylprednisolone versus placebo in IgAN. The study includes patients with a wide range of eGFR values, from 20 to 90 ml/min per 1.73 m ^2^.

***Cyclophosphamide in combination with corticosteroids.*** In Caucasian subjects, cyclophosphamide plus corticosteroid therapy may benefit patients at high risk of developing ESRD, namely those with glomerular crescents and rapidly progressive clinical course ^34, 35^. In Chinese patients, crescentic IgAN carries a poor prognosis. Amongst 113 such patients from eight centers across China, no benefit was observed in the renal survival when cyclophosphamide was added to pulse corticosteroid therapy ^36^. The 2012 KDIGO Guidelines ^29^ suggest the use of corticosteroids and cyclophosphamide in patients with IgAN and rapidly progressive crescentic IgAN, analogous to the treatment of anti-neutrophil cytoplasmic antibody (ANCA) vasculitis, but this is not based on evidence from clinical trials.

***Tonsillectomy in combination with corticosteroids.*** The practice of tonsillectomy in IgAN (mainly in Japan) is based on observation of disease activation, manifested as macroscopic hematuira and renal dysfunction following upper respiratory tract infection. Recently, a gene encoding glycosyltransferase involved in glycosylation of O-glycan in IgA molecules has been demonstrated to be downregulated in tonsillar B lymphocytes from patients with IgAN ^37^. Outside Japan, the benefits of tonsillectomy have not been documented. A meta-analysis of seven non-randomized studies (mostly from Japan) comprising 858 patients (534 underwent tonsillectomy and 324 did not) showed that tonsillectomy combined with either standard or pulse corticosteroid treatment, but not tonsillectomy or corticosteroid treatment alone, resulted in higher remission rates with favorable long-term outcome ^38^. Lately, a multicenter randomized controlled trial from Japan showed that tonsillectomy combined with steroid pulse therapy has no beneficial effect over steroid pulses alone to attenuate hematuria and to increase the incidence of clinical remission ^39^. Although the anti-proteinuric effect was greater in combined therapy, the difference was marginal. Finally, a recent meta-analysis of 14 studies (also mostly from Japan) also found positive effects of tonsillectomy plus pulse or conventional steroid ^40^. The 2012 KDIGO Guidelines ^29^ suggest that tonsillectomy not be performed for IgAN.

***Azathioprine.*** A recent moderately large-scale study randomly assigned 207 patients to either corticosteroids alone (n = 106) or in combination with azathioprine (n = 101) for 6 months ^41^. Azathioprine conferred no additional benefit but resulted in more adverse events, namely hepatotoxicity, anemia, and gastrointestinal symptoms. The 2012 KDIGO Guidelines ^29^ do not recommend the use of azathioprine in IgAN.

***Mycophenolate mofetil.*** Three studies in Chinese patients showed a benefit of mycophenolate mofetil (MMF): (i) In 62 patients with severe IgAN and urinary protein of more than 2 g/day, the MMF group showed significant improvement in proteinuria and serum lipids than the prednisone group ^42^. (ii) Among 40 Chinese patients with mild tubulointerstitial lesions with persistent proteinuria of more than 1 g/day despite full RAS blockade, MMF treatment for 6 months resulted in significant reduction in proteinuria ^43^ and improved renal survival at 6-year follow-up ^44^. (iii) In a study comparing therapy with MMF/prednisone and cyclophosphamide/prednisone for severe IgAN ^45^, the former regimen achieved a higher remission rate with better reduction of proteinuria and improvement of renal function and less adverse effects.

In contrast, three studies in Caucasians showed mixed results: (i) Among 34 Belgian patients with impaired renal function, histologic unfavorable criteria and arterial hypertension, a combination of salt restriction, ACEI therapy and high-dose MMF failed to demonstrate a better beneficial effect after 3 years of evaluation ^46^. (ii) In an American study that recruited patients with even more advanced renal insufficiency, a worse outcome occurred with MMF as a “salvage” therapy ^47^. (iii) In another Italian study, a subset of IgAN patients with florid glomerular changes treated with MMF and corticosteroids showed remission of proteinuria and reversal of progressive renal failure ^48^.

Given that these mixed results occurred across different ethnic groups and that none of these studies was adequately powered to provide a definitive answer, the 2012 KDIGO Guidelines ^29^ suggest not using MMF in IgAN. More recently, one trial conducted in 52 children, adolescents, and adults with IgAN in the US and Canada was terminated prematurely as MMF did not reduce proteinuria ^49^. Patients received lisinopril (or losartan) plus a highly purified omega-3 fatty acid for 3 months during run-in, and only those with a persistent urinary albumin/creatinine ratio of at least 0.6 g/g (males) or at least 0.8 g/g (females) were randomly assigned.

### Novel therapies

Increased knowledge on the pathogenetic mechanisms of IgAN, particularly on the role of mucosal immunity and B-cell activation, has provided the impetus for several new phase II/III clinical trials. None of these have reached any conclusions yet.

***Enteric budesonide.*** NEFIGAN is a phase IIb trial that was started in 2012 to evaluate the efficacy and safety of an enteric budesonide delivered specifically to the ileocecal Peyer’s patches in primary IgAN across 10 European countries. Preliminary study demonstrated a reduction of proteinuria by 23% and a modest improvement of eGFR by 8% in 16 patients (proteinuria of more than 0.5 g/day and serum creatinine of less than 200 μmol/l) after 6 months of enteric budesonide, followed by 3 months of further observation ^50^. The study was completed in September 2015 and found encouraging results in terms of proteinuria reduction and stabilization of renal function at 9 months (ASN Kidney Week 2015).

***B-cell depletion/inhibition.*** Blisibimod is a selective peptibody antagonist of BAFF that can be administered subcutaneously. BRIGHT-SC (Blisibimod Response in IgAN Following At-Home Treatment by Subcutaneous Administration) began in 2013 and is currently recruiting patients in Asia and Europe.

***Spleen tyrosine kinase (Syk) inhibition.*** An important molecule within an intracellular signaling pathway activated upon ligation of the B-cell receptor is Syk, which mediates maturation and survival of the B-cell lineage. Pharmacological inhibition of Syk, or its knockdown by small interfering RNA (siRNA), significantly reduced cellular proliferation and the synthesis of pro-inflammatory mediators in human mesangial cells exposed to IgA1 from patients with IgAN ^51^. SIGN (Syk Inhibition for Glomerulonephritis), a multicenter trial, started recruitment in 2014 globally to evaluate the efficacy of fostamatinib (a selective oral Syk inhibitor) in patients with IgAN.

***Proteasomal inhibition.*** There is preliminary evidence for a role of increased immunoproteasome activity in IgAN ^52^. A single-center, open-label, exploratory study examining the effects of bortezomib (Velcade) in IgAN was started in 2010 in the US.

## Conclusions

Despite a better understanding of the immunochemical nature of aberrantly glycosylated IgA1 and the genetic risk profile of IgAN, the key issue of how disease can be triggered following recurrent mucosal infection remains unknown. Specific treatment is lacking. Diagnosis by a non-invasive method such as disease biomarkers without invasive renal biopsy will allow increased disease detection rate and early treatment intervention.
